# Lytic and Non-Lytic Permeabilization of Cardiolipin-Containing Lipid Bilayers Induced by Cytochrome *c*

**DOI:** 10.1371/journal.pone.0069492

**Published:** 2013-07-22

**Authors:** Jian Xu, T. Kyle Vanderlick, Paul A. Beales

**Affiliations:** 1 Department of Chemical and Environmental Engineering, Yale University, New Haven, Connecticut, United States of America; 2 Centre for Molecular Nanoscience, School of Chemistry, University of Leeds, Leeds, United Kingdom; University of South Florida, United States of America

## Abstract

The release of cytochrome *c* (cyt *c*) from mitochondria is an important early step during cellular apoptosis, however the precise mechanism by which the outer mitochondrial membrane becomes permeable to these proteins is as yet unclear. Inspired by our previous observation of cyt *c* crossing the membrane barrier of giant unilamellar vesicle model systems, we investigate the interaction of cyt *c* with cardiolipin (CL)-containing membranes using the innovative droplet bilayer system that permits electrochemical measurements with simultaneous microscopy observation. We find that cyt *c* can permeabilize CL-containing membranes by induction of lipid pores in a dose-dependent manner, with membrane lysis eventually observed at relatively high (µM) cyt *c* concentrations due to widespread pore formation in the membrane destabilizing its bilayer structure. Surprisingly, as cyt *c* concentration is further increased, we find a regime with exceptionally high permeability where a stable membrane barrier is still maintained between droplet compartments. This unusual non-lytic state has a long lifetime (>20 h) and can be reversibly formed by mechanically separating the droplets before reforming the contact area between them. The transitions between behavioural regimes are electrostatically driven, demonstrated by their suppression with increasing ionic concentrations and their dependence on CL composition. While membrane permeability could also be induced by cationic PAMAM dendrimers, the non-lytic, highly permeable membrane state could not be reproduced using these synthetic polymers, indicating that details in the structure of cyt *c* beyond simply possessing a cationic net charge are important for the emergence of this unconventional membrane state. These unexpected findings may hold significance for the mechanism by which cyt *c* escapes into the cytosol of cells during apoptosis.

## Introduction

The release of cytochrome *c* (cyt *c*) from the intermembrane space of mitochondria is a key step in the initiation of cell death (apoptosis) [1]. Cyt *c* complexes with apoptotic protease-activating factor 1 (Apaf-1) in the cytosol to form the apoptosome and trigger activation of “executioner” (caspase) proteins that then proceed to breakdown the cell’s protein machinery from the inside. Despite its significance, the mechanism by which cyt *c* is released from mitochondria is not fully understood and is often simply referred to as mitochondrial outer membrane permeabilization (MOMP) [1], [2].

During normal cell function, cyt *c* has high affinity for cardiolipin (CL) lipids in the inner mitochondrial membrane (IMM) and transfers electrons between complexes III and IV of the electron transport chain during the production of ATP, the cell’s primary source of chemical energy [2]. During intrinsic apoptosis, often triggered by oxidative stress, the association between CL and cyt *c* is thought to weaken and pores form in the outer mitochondrial membrane (OMM) that allow cyt *c* and other small proteins that reside within the intermembrane space to escape into the cytosol [3]. Anti- and pro-apoptotic Bcl-2 family proteins, e.g. Bak and Bax, are known to regulate the release of cyt *c* from mitochondria, but their exact mechanisms are not yet clear [2]. The proapoptotic Bcl-2 family proteins may oligomerize to form pores in the OMM, cause widening of VDAC channels in the OMM to form large pores for cyt *c* to escape, or have alternative interactions that ultimately result in MOMP. Significant morphology changes are also known to occur in the mitochondrial membranes during apoptosis [4] that lead to a substantial increase in the CL content of the OMM (from ∼4% during normal function to >20% during apoptosis) [3], [5], [6], [7].

Previous investigations of CL – cyt *c* interactions in giant vesicle model systems have shown that, under certain conditions, fluorescently labeled cyt *c* and passive leakage markers of a similar size (10 kDa fluorescent dextrans) cross the membrane into the interior lumen of the vesicles [8]. These observations demonstrate the stochastic formation of nanoscale pores in the membrane induced by the CL – cyt *c* interaction. CL-rich (>∼75%) lipid systems are also known to form a non-bilayer H_II_ phase in the presence of cyt *c*: this lyotropic phase is characterized by an inverted micellar structure with hexagonally-packed aqueous channels running through it [9], [10]. The formation of these porous structures is a consequence of the small hydrophilic headgroup and bulky hydrophobic tails of CL lipids that result in a molecular shape that prefers to pack in regions of membrane with a negative curvature, i.e., where the lipid monolayer bends towards the aqueous phase. Therefore a packing stress occurs in lipid bilayers where such lipids are constrained to form part of a flat membrane, which can increase the membrane’s susceptibility to undergo conformational changes in response to external stimuli [11], [12], [13]. The potential importance of these observations for cyt *c* release from mitochondria during apoptosis is clear: if cyt *c* itself can induce holes in CL-containing membranes that enable these proteins to permeate across these barriers, then this is a possible mechanism that biology could utilize for MOMP. Previous observations of macromolecular permeation across giant vesicle membranes have the disadvantage of not allowing long time kinetic observations of membrane permeability: once the vesicles have fully leaked to the fluorescent probes, no further evolution of trans-membrane transport can be detected [8]. Therefore we now explore this permeation phenomenon in further detail using a minimal model that allows us to investigate the parameters that affect the permeability of these membranes under physiologically relevant conditions over extended periods of time to gain greater insight into the pore formation mechanism and their temporal evolution.

**Figure 1:**
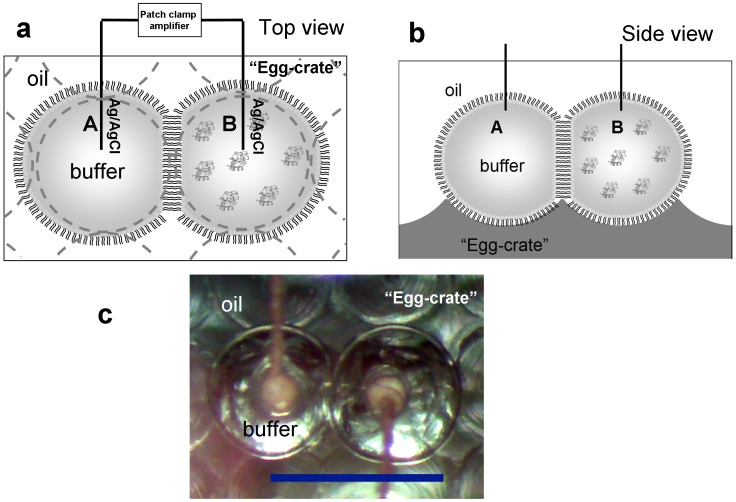
Schematic of the nanoliter drop bilayer setup: a) top view; b) side view (not drawn to scale). The left droplet contains buffer only, while the right droplet also contains cyt *c*. A voltage is applied across the droplet bilayer, with the right droplet is grounded. Pore formation is monitored by recording the ionic current through the bilayer membrane. c) Actual top view under the microscope: scale bar  = 1 mm.

Our investigations of this model mitochondrial membrane system take advantage of the nanoliter droplet interface bilayer formation method [14], [15], [16], a relatively new and powerful technique which allows detection of pore formation in lipid bilayers by electrophysiology and to simultaneously monitor membrane integrity/stability *via* optical microscopy. The experimental set-up is shown in Figure 1. Aqueous droplets (∼200 nL, approximately 700 µm in diameter) are first formed in hexadecane containing dissolved lipids which form a stabilizing monolayer at the water/oil interface. Electrodes, which are readily inserted into individual droplets, are used to bring two droplets together thus creating a lipid bilayer between them and allowing current measurements across the membrane under applied voltages. The droplets are nestled within an “egg crate” platform (an array of micro-indents was manufactured by computer-aided electric drills with a separation of 700 µm on a poly(methyl methacrylate) plate, Figure 1b) to facilitate their handling.

The attributes of this novel technique are critical to this study, which reveals an anomalous, highly permeable membrane state that is mechanically stable. In the droplet bilayer formation method, the membrane permeability and the membrane integrity can be observed independently: the membrane permeability is monitored by the electrophysiological measurement of membrane ionic current; the membrane integrity is directly visualized by the configuration of droplets (whether the two droplets merge) and is further verified by the disassembly and reassembly of droplet bilayer via moving droplets closer or further away. This anomalous non-lytic but highly permeable state would have been missed if traditional bilayer fabrication schemes were employed, such as the painting method [17], [18] and Montal-Mueller technique [18], [19], [20], in which membrane integrity is often indirectly monitored through the membrane permeability by the electrophysiological measurement of ionic current through the membrane: the membrane is inferred to be ruptured when the current through the membrane is overloaded [18], [20], [21].

## Results

As our basic system, the nanoliter droplet method was used to create bilayers composed of the planar bilayer forming lipid 1,2-diphytanoyl-sn-glycero-3-phosphocholine (DPhPC), cholesterol, and CL in molar ratios of 55∶25∶20. This CL level is similar to that found in inner mitochondrial membranes [5], [22] and outer mitochondrial membranes under apoptotic conditions [3]. The pH of the aqueous droplets was set at 7.4, similar to physiological conditions. Unless otherwise noted the aqueous buffer forming the droplet interiors contained 200 mM KCl and 10 mM MOPS. One of the two abutting droplets was formed in buffer with added cyt *c* (at various concentrations). Electric current across the membrane was measured upon applied voltages of −50 mV.

**Figure 2:**
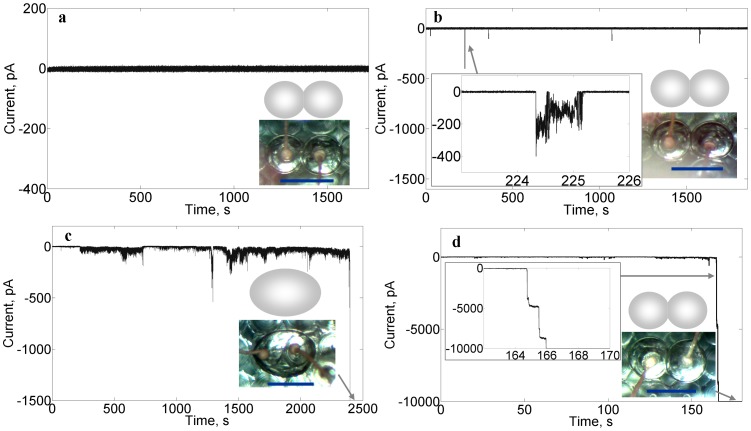
Membrane current responses at various concentrations of cyt *c* (a, 507 pM; b, 84.7 nM; c, 254 nM; d, 2.54 µM). Each response is characteristic of a different state of permeabilization: a) impermeable; b) porous, non-lytic; c) porous, lytic; d) highly permeable non-lytic. Inset microscopy images show the state of droplets (unmerged in a,b,d; merged in c); a cartoon image is shown above each microscopy image for further elucidation of the droplet configurations. Inset graphs are detailed images of the current at indicated periods of time. Each droplet contains 200 mM KCl, 10 mM MOPS, pH 7.4 buffer; membrane composition is DPhPC/chol/CL  = 55%/25%/20%; and voltage clamp: −50 mV.

Current measurements clearly show that the membranes become permeable as the concentration of cyt *c* increases above ∼10^1^ nM, at which point electrical spikes occur on the otherwise current-free background (see Figure 2a & 2b). The spikes vary in amplitude from 10^1^∼10^2^ pA. As the concentration of cyt *c* is raised, the frequency of spikes increases, as shown in Figure 2c. Eventually a steady-state (overloaded) current is reached which exceeds the measurement range (−10000 pA) and this occurs concomitantly with membrane lysis and a merging of the two droplets into one, as seen under the optical microscope.

The presence and nature of electrical spikes indicate that ion permeability across the lipid membrane occurs via transient pore formation. The size of the pores can be estimated from the amplitude of these electrical spikes according to a simple electrolyte conductance model [23], [24]:
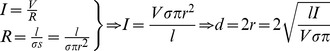
(1)Here, *r* and *d* are the calculated average radius and diameter of the pore, *I* is the measured ionic current, *V* is the applied voltage, *R* is the resistance of the lipid membrane with pores formed, *l* is the lipid membrane thickness (estimated to be ∼4 nm [17], [25], [26]), and σ is the conductivity of the electrolyte (measured as 24.85 mS·cm^−1^). The amplitude of electrical spikes observed ranges from 50 to 400 pA, yielding pore diameter estimates ranging between 1.4 nm and 4.0 nm. The transient pore formation of this state has been observed at various experimental conditions. Additional electrophysiological recording of pore traces with longer lasting period (∼10^1^ s) is presented in Figure S1 in File S1 (supporting information), which indicates formation of a ∼3.4 nm diameter pore.

It is not surprising that an increase in the frequency of pore formation, as caused by increasing the concentration of cyt *c*, ultimately leads to membrane lysis (which results in a merger of the two droplets). Membrane mechanical failure is suggested by simple energetic models [27], [28], [29], which predict that pores in close proximity will combine to form larger pores so as to reduce the total interfacial energy of the system (in this case the one-dimension line tension associated with the edge of each pore) and, once a threshold size is reached, will grow rapidly (driven by the membrane’s surface tension) leading to catastrophic failure of membrane integrity (Figure S2 in File S1).

What is surprising, however, is that our experiments also reveal, at sufficiently high cyt *c* concentrations (µM∼mM levels), an anomalous highly permeable non-lytic membrane state. As with lower cyt *c* concentrations that lead to lysis, the system first exhibits dense electrical spikes against the baseline until sufficient time has elapsed that the current increases to the overloading level (usually a few minutes, as shown in Figure 2d). Unlike the lytic concentrations, however, these higher-dosed cyt *c* droplets do not merge. On the contrary, the bilayer remains fully intact, all the while permitting massive ion fluxes, as shown in Figure 2d.

**Figure 3:**
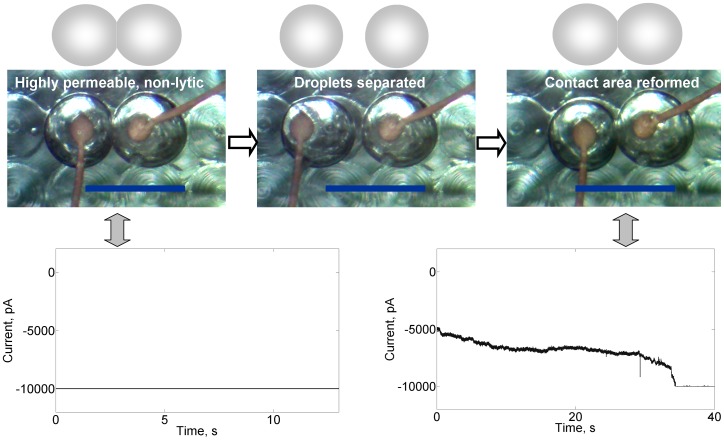
Reversibility of the highly permeable non-lytic membrane state which occurs at high cyt *c* concentrations. Microscopy and cartoon images are shown in the top panels; for clarity, the cartoons show the droplet configurations seen in the images. Current (bottom panels) across the intact bilayer can be halted by physically separating the droplets. Current is reestablished (to the overloading level) when the droplets are brought back together to reform the permeable bilayer. Buffer: 200 mM KCl, 10 mM MOPS, pH 7.4; membrane composition: DPhPC/chol/CL  = 60%/25%/15%; cyt *c*: 25.4 µM; voltage clamp: −50 mV.

Remarkably, the highly permeable lipid bilayer at the interface of two droplets (Figure 3, top left panel and bottom left panel) can be dissembled into two monolayers by mechanically detaching the droplets through moving the left electrode away, which moves the left droplet away accordingly and shuts down the ion exchange pathway between the aqueous compartments (Figure 3, top middle panel). However, once the lipid bilayer is reassembled by mechanically abutting the two droplets (Figure 3, top right panel) through moving the left electrode back to its original position, the membrane permeability is quickly established (<40s as shown in Figure 3, bottom right panel). One can repeatedly dissemble and reassemble the droplets, each time reestablishing enormous ion transport when the bilayer is formed. Moreover, the temporal robustness of this unique highly permeable non-lytic membrane state is extraordinary, persisting for at least 20 h (the maximum duration tested).

**Figure 4:**
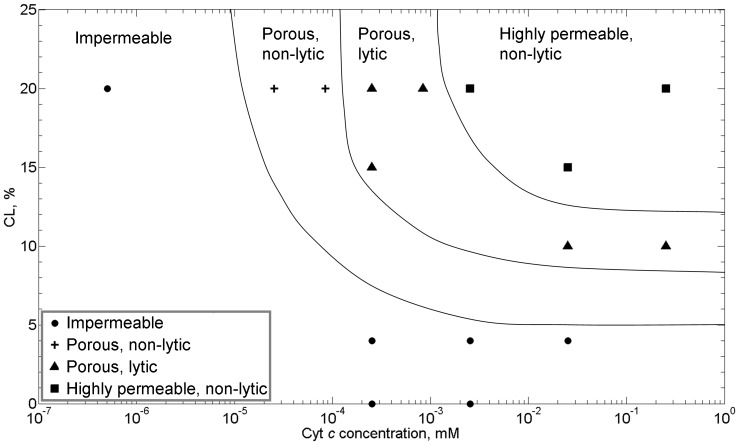
Permeability “map” of the system as a function of cyt *c* concentration in solution and cardiolpin concentration in the membrane. Different states of permealization are delineated: • impermeable;+porous, non-lytic; ▴ porous, lytic; ▪ highly permeable, non-lytic. Lines are guides to eye delineating the various states. Buffer: 200 mM KCl, 10 mM MOPS, pH 7.4; voltage clamp: −50 mV. All the lipid membranes contain 25% chol, except that the 0% CL lipid membrane doesn’t contain any chol. Each data point in the permeability “map” is deduced from a miniumum of 5 independent experiments.

A “permeability map” for these CL-containing bilayers can be created, as shown in Figure 4, reflecting the different membrane states observed – impermeable, porous/non-lytic, porous/lytic, and the anomalous highly permeable non-lytic state – as a function of both cyt *c* and CL concentrations. We note that control experiments show that both cyt *c* and CL are needed for the system to exhibit pore formation. With both present, increasing the concentration of either molecule makes the membrane more permeable. As a corollary, the transitions between membrane permeabilization states occur at lower concentrations of cyt *c* as the concentration of CL in the membrane is increased.

Previous studies suggest that electrostatic interactions govern/regulate the interaction of the positively charged cyt *c* with membranes containing the negatively charged CL [30], [31], [32], [33]. To probe this further, we examined the influence of ionic strength on the permeability behavior of these membrane systems by varying salt concentrations from 50 mM to 3 M KCl. Membrane CL concentration was fixed at 20%. As shown in Figure 4, increasing the ionic strength suppresses pore formation, shifting the different permeability states to higher concentrations of cyt *c*. The presence of the anomalous highly porous non-lytic membrane state persists (but is shifted to higher levels of cyt *c*). The observed changes in permeability are consistent with the notion that electrostatic interactions are important in these systems and can be modulated, i.e., screened, by adjusting ionic strength (with higher salt concentrations leading to increased screening).

To further explore the significance of electrostatic interactions in regulating the permeability of these model mitochondrial membranes, we performed nanoliter droplet experiments replacing cyt *c* with similarly charged polymer particles, in particular two different PAMAM dendrimers. These “tree-like” amine-terminated branched polymer molecules (which are synthesized in “generations”) are positively charged, and are often used as artificial mimics for natural spherical proteins [34], [35]. For these studies we tested both Generation 2 particles which have a radius of 1.3 nm, 16 surface amine groups and a molecular weight of 3.26 kDa, and Generation 3 particles with a radius of 1.8 nm, 32 surface amine groups and a molecular weight of 6.91 kDa [34], [35]. For comparison, the radius of cyt *c* (which is ∼12 kDa in molecular weight) is reported to be between 1.5 and 1.7 nm [36], [37], and its approximate charge at physiological pH is ∼+8*e* [38], [39].

PAMAM G2 and G3 dendrimers at the concentration range from 0 to 250 µM have been examined in the CL-containing bilayer system at the similar experimental conditions as the cyt *c* (please see the Supporting Information File S1 for detailed discussion and Figure S3 in File S1). In short, we find that the PAMAM dendrimers also induce pore formation as revealed by the electrical measurements, including sufficient pore formation to cause membrane lysis (and droplet merger) at high enough PAMAM concentrations. In fact, relative to PAMAM, larger concentrations of cyt *c* are needed to cause the same degree of permeability and lysis. For example, 25 nM of cyt *c* is not sufficient to cause membrane lysis, which does occur for equivalent concentration of the PAMAM G2 and G3.

Most interestingly, we were unable to observe a corresponding anomalous high permeability non-lytic membrane state with this polymer perturbant (even though, as discussed above, both generations of PAMAM permeabilize the membrane more readily than cyt *c* at equivalent concentrations). Increasing the PAMAM concentration up to ∼10^2^ µM (Figure S3 in File S1) continues to lead to membrane lysis (by way of comparison, at 25 µM cyt *c* the membrane is in the anomalous highly permeable non-lytic state, Figure 4).

## Discussion

### (i) Proposed Physical Mechanisms of Membrane Permeabilisation

The current flow across the CL-containing bilayers, as reflected by the electrical measurements, demonstrate transient pore formation in the membranes induced by sufficient exposure to cyt *c*. As reported above, the pore sizes are not fixed but fluctuate randomly between 1.4 nm and 4.0 nm. While the exact nature of these pores is not obvious, they are most likely transient lipid pores, where the variation in pore size reflects the fluidity of lipid assemblies and the absence of a tightly defined pore size and structure as would be expected for protein-based membrane channels, whether static [40], [41] or gated [42], [43]. While it is possible that cyt *c* (the only protein present in our system) could itself be a pore former this is unlikely given the globular nature of this molecule.

**Figure 5:**
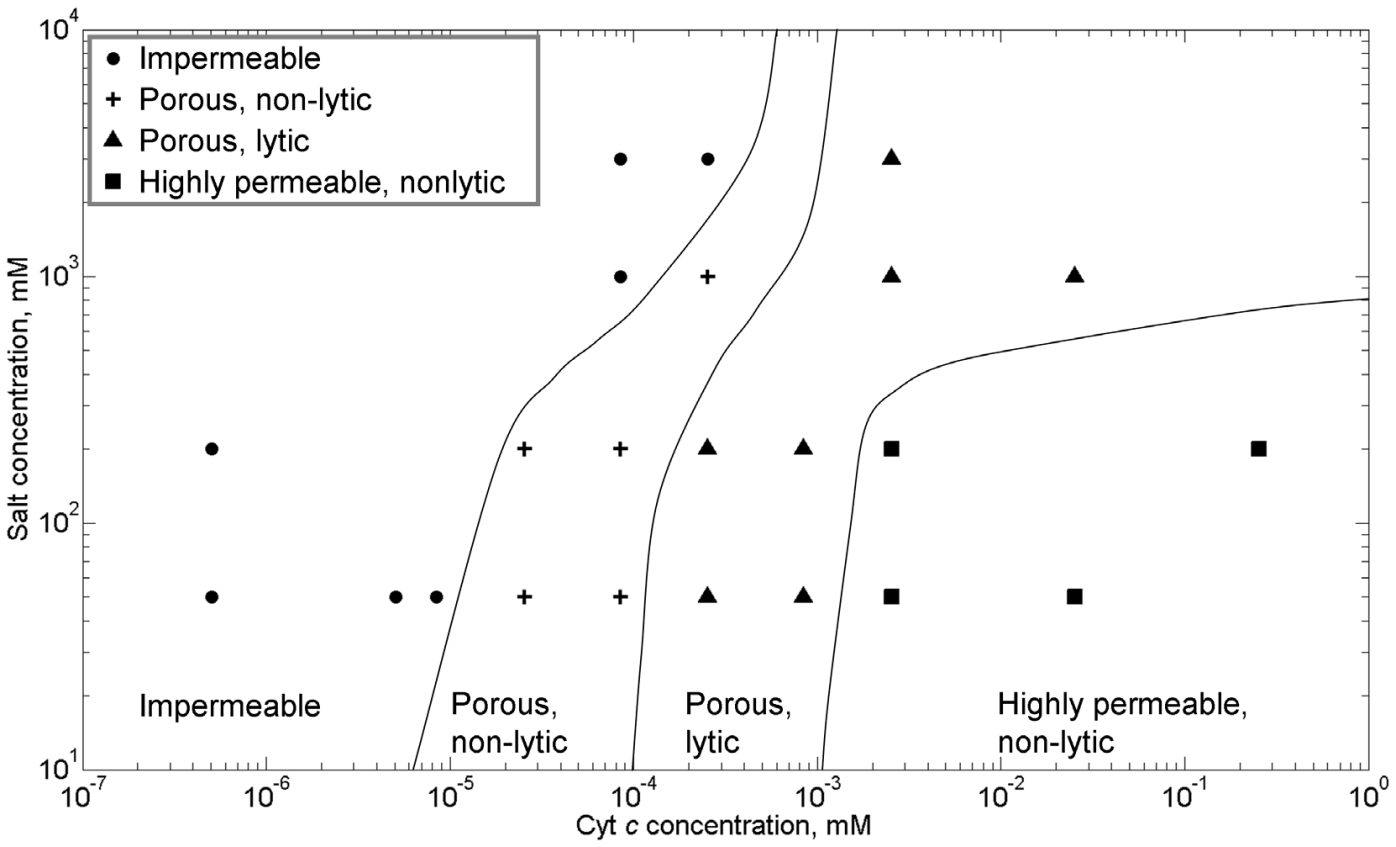
Influence of ionic strength on states of permeability: • impermeable;+porous, non-lytic; ▴ porous, lytic; ▪ highly permeable, non-lytic. Lines are guides to eye delineating the various states. Membrane composition: DPhPC/chol/CL  = 55%/25%/20%. Each data point in this figure is deduced from a minimum of 5 independent experiments.

It is clear that the mechanism for pore formation has some origin in the electrostatic interactions of the key protagonists: CL and cyt *c*. The transitions between permeabilisation regimes are strongly dependent on the concentrations of both CL and cyt *c* (Figure 4), which carry electrical charges of opposite polarity, and these transitions are significantly suppressed by the increased screening of electrostatic interactions from the increased presence of electrolyte (Figure 5). Furthermore, replacement of cyt *c* with other cationic, globular polymers (PAMAM dendrimers) of similar hydrodynamic radii also resulted in qualitatively similar, dose-dependent pore formation in the membrane. Previous studies have shown that electrostatic interactions from cationic proteins can induce the local clustering of anionic lipids within a membrane [33]. Local clustering of anionic CL lipids, which prefer structures with negative spontaneous curvature, would increase the local curvature stress in the membrane by bringing the local composition closer to that of a L_α_ – H_II_ transition [9], [10], and thereby likely increasing the propensity for dynamic structure changes (e.g. pores) to transpire in the membrane. Indeed, a detailed mathematical model for the mechanism of cyt *c* induced pore formation in CL-containing membranes has recently been proposed: electrostatic clustering of CL under cyt *c* induces a local curvature stress in the membrane, which decreases the curvature elastic energy for formation of a finite-sized, toroidal lipid pore approximately 2 nm in diameter [8].

Lipid pores have previously been reported for membranes close to their melting transition temperature (T_m_). These lipid pores may spontaneously form and resemble certain electrophysiological features of protein pores [44]. However, at temperatures far from the T_m_, any lipid pores that form in membranes are known to fluctuate in size and eventually reseal due to the fluidity and dynamic nature of lipid molecules within the bilayer [18], [45]. In our current work, the membranes are far (>20°C) above the T_m_ of these lipid mixtures (T_m_ (DPhPC)< −20°C [46]; T_m_ (CL) <6°C [47]) and therefore well outwith the regime for pore formation at the chain melting transition.

The initial dose-dependence of membrane permeability on cyt *c* concentration, up to the lytic regime, is consistent with the behavior reported for other perturbants. A threshold concentration of perturbant is required before ion permeability is measured through the membrane. On increasing concentration further, pore formation in the membrane increases to the point where this can no longer be sustained causing the membrane to rupture. Qualitatively, this behavior is consistent with the action of other pore-forming proteins and peptides, such as Bax [48], [49], melittin [50], [51] and a peptide fragment from a human immunodeficiency virus (HIV) transmembrane protein gp41 [52]. This generic lytic phenomenon, observed across a variety of perturbants, is consistent with the behavior predicted by the free energy model for pore formation and membrane rupture, previously discussed in the results section (Figure S2 in File S1). If pores grow or merge to a size beyond a threshold radius (predicted to be ∼5 nm for a typical lipid bilayer), pore growth will rapidly and uncontrollably increase such that the membrane destabilizes and lysis occurs.

The extraordinary discover here is that, beyond the lysis regime, we find a reentrant non-lytic state characterized by extremely high ion permeability. This state has high temporal stability (>20 h) and the monolayers of the droplets can stably and reversibly be pulled apart and the bilayer reformed with the highly permeable state quickly reestablishing itself (within a few min.). The mechanism for formation of this non-lytic highly permeable state is not yet fully understood. However, based on the observed features of this state, the reported dynamics of pore formation and membrane stability [27], [28], [29], and the known characteristics of the interaction of cyt *c* and lipid membrane [8], [9], [10], [53], we propose a plausible mechanism that explains our current observations. Given our previous discussion of a critical pore radius being required before lysis occurs, we assume that a size-limiting mechanism now prevents pores reaching this critical size. The origin of this size-limiting mechanism is unclear. However we reasonably assume that this is a result of repulsion between individual pores within the membrane, preventing them from coalescing to minimize their line tension, and/or restriction of the growth of individual pores.

An electrostatic origin for this size limiting mechanism is again plausible because we observe suppression of the non-lytic, highly permeable state at high salt concentrations (Figure 5). However, this non-lytic regime was unique to cyt *c* in our experiments, i.e., it was not observed for perturbation by the PAMAM dendrimers, which are more membrane active than cyt *c*, causing membrane permeation at lower molar concentrations. This could be a result of higher charge densities on PAMAM dendrimers (similar size to cyt *c* but higher net surface charge) or as a result of other more specific, non-ionic interactions between cyt *c* and PC/CL membranes [54], such as conformational changes in cyt *c*, e.g. unfolding of the C-terminal helix, that might stabilize the pore structure [8]. It is also interesting to note that, since samples composed of high CL and cyt *c* concentrations are known to form a highly porous, inverted hexagonal H_II_ phase [9], [10], [53], it could also be speculated that the lipid interface between abutting droplets in this non-lytic, highly permeable state might be a quasi-2D analogue of this inverted lyotropic liquid crystal phase (or other inverted, e.g. cubic, lyotropic phase). Intriguingly, formation of a cubic lipid phase has been observed in the mitochondria of amoeba [55].

### (ii) Possible Physiological Relevance of Our Findings

Comparison of our permeability measurements to physiological conditions of native mitochondria is consistent with behavior in normal and apoptotic states. At a physiological salt concentration of 125 mM KCl and normal OMM CL composition of 4% [5], [22], no permeabilization of the membrane is observed across all concentrations of cyt *c*, including those corresponding to the concentration range of cyt *c* reported to be found in the mitochondrial inter-membrane space (300–700 µM) (Figure 4) [56]. However, during apoptosis the CL content of the OMM is known to significantly increase up to compositions as high as ∼40% due to conformational changes in the mitochondrial membranes that transfers CL from the inner to the outer membrane [3], [6]. At these higher CL concentrations, our data would predict that the membrane would now be well within the non-lytic, highly permeable regime. It is also of note that the size of pores calculated from peak current measurements in the transient pore regime (1.4–4 nm) appear to be large enough for cyt *c* (3–3.4 nm) to pass through them, consistent with recent quantitative observations of the macromolecular permeability of CL containing membranes in the presence of cyt *c* [8].

The exact nature of the MOMP transition that permits cyt *c* escape from the mitochondrial inter-membrane space into the cytosol is still to be confirmed [57], [58], [59]. Until fairly recently it was unclear whether the OMM ruptured during apoptosis [57], [58], [60], but further studies now seem to agree that the OMM remains intact [36], [58], [59], [61], [62]. The current consensus in the literature appears to work on an underlying assumption that these pores are formed by proteins. However patch clamp studies on OMM extracts from apoptotic cells have shown variable pore diameters in the range 2.9–7.6 nm [36]. These observations of variable pore sizes within this range are consistent with the possibility that these pores are in fact lipidic in nature. Interestingly, cells can sustain small amounts of “background” leakage of cyt *c* from mitochondria during normal function [2]; this behavior may be analogous to the transient pore regime observed in our model system.

The relevance of these results to the mechanisms of MOMP formation during the onset of apoptosis in cells will require further work to fully establish. Other proteins have been implicated to have pro- and anti- apoptotic effects relevant to the release of cyt *c* into the cytosol, in particular the Bcl-2 family proteins, e.g. Bak and Bax [63], [64], [65]. The droplet bilayer system we use here would likely be an ideal experimental set up to investigate the impact of these proteins on the pore-forming behavior of cyt *c* in a minimal model system with exquisite control over the competing experimental variables. Several hypotheses are plausible based upon our current findings: (1) the role of these pro/anti-apoptotic factors could be in the regulation of mitochondrial membrane morphology changes that lead to the significant increase of CL content in the OMM; (2) cyt *c* induced permeabilization of CL-rich membranes may not be the sole mechanism for MOMP, but is one of several parallel mechanisms for instigation of apoptosis in cells due to the high importance of efficient cell death for the healthy life-cycle of organisms; (3) cyt *c* mediated membrane permeabilization could be an evolutionary relic of a primitive form of apoptosis that is no longer utilised by nascent life; or (4) a possible pathway that nature never took advantage of. Much further work, including cell studies, will be required to underpin the relevance of these findings with regards the multifarious interactions at the OMM of apoptotic cells.

## Summary

Using the innovative droplet bilayer system for simultaneous electrochemical and microscopy measurements, we have clearly demonstrated that cyt *c* can induce permeabilization of CL-rich membranes as a result of electrostatic interactions. The activity of cyt *c* is dose dependent such that the membranes are impermeable at low concentration, while transient pore formation occurs in the nanomolar regime (10 − 100 nM) until membrane lysis eventually results for concentrations in the range 0.1–1 µM. The astounding discovery is that at concentrations beyond a few µM cyt *c*, a non-lytic, highly porous state is found to occur with the membrane forming a stable but highly permeable barrier between aqueous compartments. The conditions under which we find this non-lytic, highly permeable state in this minimal model are comparable with physiological conditions of mitochondria in an apoptotic cell. Therefore these results suggest that after conformational changes in the mitochondrial membranes that increase the CL content of the OMM and release of cyt *c* tightly bound to CL on the IMM, cyt *c* may itself be capable of punching holes in the OMM (i.e., MOMP) that enable it to escape into the cytosol, activating a downstream signaling cascade leading to cell death.

## Materials and Methods

Lipids, including 1,2-diphytanoyl-sn-glycero-3-phosphocholine (4ME 16∶0 PC, DPhPC), cholesterol (chol) and cardiolipin (heart, bovine) (sodium salt), were purchased from Avanti Polar Lipids Inc. Cytochrome *c* from *Saccharomyces cerevisiae* and hexadecane (oil) were purchased from Sigma-Aldrich. Potassium chloride (KCl) and 3-(N-morpholino)propanesulfonic acid **(**MOPS) were purchased from Alfa Aesar. PAMAM dendrimers were purchased from Dendritech Inc. All the buffers were made from deionized (DI) water.

The lipid bilayer is constructed at the interface of two nanoliter droplets [14], [15], [16]: lipids, usually containing DPhPC, chol and CL with appropriate ratios, are premixed in oil (hexadecane) and vortexed on a digital vortex mixer (VWR) at 3000 rpm for 5 min. to form a 10 mM lipid solution in oil; two aqueous droplets (200 nL each) are suspended in the oil/lipid mixture; each droplet contains the KCl solution in MOPS buffer and the pH of the solution is adjusted to ∼7.4; various concentrations of proteins are added into one certain droplet so that the direction of protein interaction with planar bilayer is defined. A lipid monolayer assembles at the droplet interface; when the two water droplets are brought into contact, a lipid bilayer forms at the interface of the two water droplets.

Although rarely found in mammalian biomembranes [22], DPhPC is a lipid with branched hydrocarbon chains that has been found in archaebacterial and extremophile membranes [66], [67]. The structure with branched saturated hydrocarbon chains make DPhPC less susceptible to photo-oxidation than unsaturated linear lipids [67], [68], while maintaining low transition temperature [69], [70]; these properties make it ideal to form artificial bilayer in the most biologically relevant fluid phase [68], [69]. In addition to the high bilayer stability, it also has low ion leakage [68], [70], which makes it an ideal model membrane for the electrophysiological study of peptide/protein-lipid interactions [14], [18], including the apoptosis regulator proteins in the mitochondrial outer membrane permeabilization (MOMP) [71], [72], [73]. The choice of lipid in this specific bilayer fabrication technique is also limited by the solubility of lipids in hexadecane; lipid in hexadecane concentrations needs to be high enough to readily form leakage free bilayers. Most of the reported droplet bilayer systems are formed by DPhPC [16], [74], [75]; many other lipids, such as POPC and DPPC, have been tested but cannot be dissolved in hexadecane in sufficient concentrations (∼ 10 mM) to form a leakage free bilayer.

The ionic current through the lipid membrane, under given applied voltage, was recorded by an Axopatch 200B patch clamp amplifier (Molecular Devices); the membrane integrity was monitored by a precision stereo zoom binocular microscope (PZMTIV-BS) on boom stand and the real-time images were taken by a digital microscope camera (DC2000M); both equipments were purchased from World Precision Instruments, Inc. Two 100 µm thick plain Ag/AgCl electrodes, held on micromanipulators, were inserted into the aqueous droplets (Figure 1 a and b) so that the ionic flow in the solution can be converted into electronic flow in the solid circuit; by applying a given voltage across the lipid membrane, the ionic current through the bilayer can be recorded by the external patch clamp amplifier to detect pore formation. Typically a negative voltage (−50 mV) is applied across the membrane to facilitate the motion of cyt *c* to the lipid bilayer, as the cyt *c* carries ∼+8*e* at neutral pH [38], [39]. A minimum of five independent experiments were conducted under each condition tested. Similar permeabilization behavior was observed at +50 mV (e.g. Figure S1 in File S1).

## Supporting Information

File S1 (DOC)
