# Supplementary material for: Lytic and Non-Lytic Permeabilization of Cardiolipin-Containing Lipid Bilayers Induced by Cytochrome c
Source: PLoS One. 2013 Jul 22;8(7):e69492. doi: 10.1371/journal.pone.0069492 (PMC3718682; doi:10.1371/journal.pone.0069492)
Supplement: File S1 — (DOC) [file pone.0069492.s001.doc]

# Supporting Information

**Lytic and Non-Lytic Permeabilization of Cardiolipin-Containing Lipid Bilayers Induced by Cytochrome *c***

Jian Xu^1^, T. Kyle Vanderlick^1^ and Paul A. Beales^2,*^

^1^ Department of Chemical and Environmental Engineering, Yale University, New Haven, CT 06511, USA

^2^ Centre for Molecular Nanoscience, School of Chemistry, University of Leeds, Leeds LS2 9JT, UK

^*^ Corresponding author: [p.a.beales@leeds.ac.uk](mailto:p.a.beales@leeds.ac.uk); Tel.: +44 (0)113 343 9101.

## Properties of Cytochrome *c* and Cardiolipin

Cyt *c* is a peripheral membrane protein to the inner mitochondrial membrane (IMM) of the mitochondrion , with ~+8e charges at neutral pH (the isoelectric point of cyt *c* is ~ pH 10) and a diameter of 3-3.4nm . Cyt *c* plays critical roles in the electron transport chain: cyt *c* transfers electrons from Complex III to Complex IV, which uses the electrons to reduce oxygen molecules to water . In addition, cyt *c* is also a key intermediate in cell apoptosis . During cell apoptosis, cyt *c* in the mitochondrion is released to the cytosol via various channels in the mitochondria outer membranes ; the released cyt *c* interacts with IP3 receptor (IP3R) of the endoplasmic reticulum (ER) and cause the ER calcium release to a cytotoxic level ; the released cyt *c* also activates a major apical cysteine protease, caspase-9, which in turn activates other proteases, such as caspase-3 and caspase-7 ; these “executioner” proteases degrade the critical protein components and destruct the cell from the inside . These channels, which release cyt *c* from the mitochondria to the cytosol, were traditionally considered to be controlled by B-cell lymphoma 2 (Bcl-2) family proteins . Under cell apoptotic conditions, pro-apoptotic Bcl-2 family proteins, such as Bax and Bak, may be oligomerized to form sufficiently large pores to facilitate cyt *c* permeation through the outer mitochondrial membrane ; some pro-apoptotic Bcl-2 family proteins may also directly bind to some channels, such as voltage-dependent anion channels (VDACs), and form large enough pores to release cyt *c* ..

Cardiolipin (CL) is a key component of mitochondrial lipid membrane . It constitutes 18-25% of the lipid in the inner mitochondrial membrane, and 4% of the lipid in the outer mitochondrial membrane (OMM) ; CL also comprises high percentages (20-24%) of lipid in mitochondrial contact sites between inner and outer membranes . In addition, during cell apoptosis, the CL composition of OMM can be increased up to 40%, due to conformational changes within mitochondrial membranes , which transfer CL from IMM to OMM . CL is a diphosphatidylglycerol lipid, which may potentially carry two negative charges per molecule; however, due to the different levels of acidities of the two phosphates (with acid dissociation constants of <4.0 and >8.0 respectively), each CL molecule only carries one negative charge at neutral pH . Since cyt *c* and CL carry reversed charges, cyt *c* has been found to associate with CL-containing lipid membranes, via electrostatic interactions . Cyt *c* was also reported to induce the hexagonal H_II_ phase, an inverted micelle structure, in CL-containing lipid membranes .

## The Nanoliter Droplet Bilayer System and Its Advantage over the Traditional Planar Lipid Bilayer Systems

In order to probe pore formation in the lipid bilayer and monitor membrane stability, we constructed a nanoliter droplet bilayer system : two aqueous droplets (~200nL) are suspended in an oil/lipid mixture; due to hydrophobic interactions, a monolayer of lipid is self-assembled to the surface of each water droplet. When two water droplets are brought into contact, a lipid bilayer forms at the interface between the droplets (main text Figure 1a). In traditional planar bilayer fabrication schemes, such as painting and folding methods, determination of the integrity of the bilayer relies upon indirect observation, i.e. whether the current through the lipid bilayer is overloading ; unlike those traditional planar bilayer schemes , the droplet bilayer method provides a direct way of monitoring membrane stability: if the lipid bilayer remains, the presence of the bilayer prevents the two water droplets (observed from the stereo microscope, main text Figure 1b) from merging. On the contrary, if the lipid bilayer at the interface of droplets ruptures, the two water droplets merge into one. Compared to the painting and folding methods , the droplet lipid bilayer system provides unique advantages of simultaneously measuring the ionic current through the lipid bilayer, to detect the formation and properties of pores, and monitoring the membrane stability by direct imaging. Because of these advantages, we discover an extraordinary “nonlytic superporous” stage in the cyt *c*-induced pore formation in the CL-containing lipid membrane, which allows large ion permeation through the membrane while maintaining its integrity. This stage would not have been discovered if the traditional painting or folding methods were employed : the overloading current through the membrane would be conventionally interpreted as membrane rupture . In addition, the nanoliter droplet bilayer system (1/200) takes only 1/5000 solution of what the traditional painting or folding method usually takes (~1ml). Therefore a higher concentration of cyt *c* is more easily obtained in the nanoliter droplet bilayer system than in the traditional painted or folded bilayer system , which is also a critical factor in detecting the extraordinary “stably porous” stage because this stage would not occur until significantly high concentration of cyt *c* is introduced to the bilayer.

## Additional electrophysiological recording of transient pore formation

We have recorded transient pore formation in CL-containing membranes in the non-lytic state, induced by cyt *c*, at various experimental conditions. The calculated pore size fits the estimated pore size range (1.4- 4.0 nm, main text). For instance, in the droplet bilayer formed between two buffer solutions of 50 mM KCl, 10 mM MOPS, pH 7.4 (the right droplet also containing 84.7 nM cyt *c*), when a +50 mV voltage clamp is applied across the membrane, transient pores of longer lasting period (~10^1^ s) are induced by the interactions between cyt *c* and CL (Figure S1). The electrophysiological recording shows the ionic current through the pore to be ~80pA. The pore size can be calculated from the same electrolyte conductance model in the main text (Eq. 1).

The conductivity σ of the buffer at this concentration was measured as 7.07 mS·cm^-1^. The calculated pore size is 3.4 nm for the transient pores observed in this period, which is within the range (1.4-4.0 nm) in the main text.

## The Dynamics of Pore Growth and Its Impact on the Stability of Lipid Bilayer

The mechanism for formation of the “nonlytic superporous” state is related to the dynamics of pore growth in the lipid bilayer and its relationship to membrane rupture. The formation of a pore will not necessarily lead to rupture of the lipid bilayer, but the formation of a large enough pore will. Generally, in any lipid bilayer, there are occasionally pores formed in the lipid membrane due to thermal fluctuations (from Brownian motion of water) or external interactions, e.g. by external electric field or by interactions from external proteins, such as cyt *c* here. The free energy to form such a pore (with radius *r*) in the lipid bilayer is determined by :

$E=2\pi\lambda r-\pi\sigma r^{2}$ (Eq. S1)

where λ is the energy cost to form a pore per unit length, which is the line tension (at the pore perimeter) and σ is the lipid bilayer surface tension. The first term is related to the energy required to break the intermolecular forces between lipids and form a circular pore edge; therefore, it is proportional to the pore circumference 2πr. The second term is the energy gained by the broadening of the pore due to the surface tension σ. These two parts of energy together determine the tendency of the pore to grow further or shrink to re-seal the bilayer.

As seen from the relationship of the free energy and the pore size (Figure S2), the free energy of the pore has a maximum at:

$\left.\frac{dE}{dr}\right|_{r=r_{c}}=0\Rightarrow r_{c}=\frac{\lambda}{\sigma}$ (Eq. S2)

The radius of the pore to obtain this maximum free energy is defined as the critical radius (r_c_) of the pore. For a typical planar lipid bilayer, λ=1×10^-11^ N and σ=2×10^-3^ N·m^-1^ , which results in a critical radius of 5 nm, i.e. 10 nm in diameter. For a pore of size smaller than r_c_, the energy reduces when the pore shrinks and the membrane becomes more stable, which is why the lipid bilayer is able to sustain small pore formation . But, when the pore size exceeds the critical size, r_c_, the pore will tend to grow, because pore growth favors the lowering of free energy, leading to rupture of the planar bilayer .

## PAMAM Interactions with CL-containing lipid membranes

Under similar experimental conditions as for cyt *c*, positively charged PAMAM dendrimers interact with CL-containing lipid membranes and cause pore formation in the lipid bilayer. PAMAM G2 and G3 dendrimers in the concentration range from 0 to 250 μM have been examined in the CL-containing bilayer system. With increasing PAMAM concentration, the multi-state pore formation phenomena was also observed. For example, ~250 nM PAMAM G3 dendrimers (Figure S3a), similar to cyt *c* (Figure 2c, main text), cause pore formation in the CL-containing lipid bilayer, eventually causing membrane rupture (lysis). However, there are significant differences between PAMAM-CL interactions and cyt *c*-CL interactions. Firstly, the concentrations of PAMAM dendrimers required to cause pore formation are lower than for cyt *c*. For example, 25 nM PAMAM (either G2 or G3) causes the porous lytic state of pore formation, while the same concentration of cyt *c* can only induce the non-lytic porous state. Secondly, there is no non-lytic highly permeable state observed for either generation of PAMAM dendrimer, under similar experimental concentrations (0 to 10^2^ μM) to that of cyt *c*. For instance, ~25μM cyt *c* induces the nonlytic highly permeable state of pore formation (Figure 4, main text), while similar concentrations of PAMAM dendrimers (Generation 3, Figure S3b) only cause the lytic porous state of pore formation. Therefore, PAMAM dendrimers can only reproduce part, but not all, of the states of pore formation exhibited by cyt *c*, within a comparable concentration range.


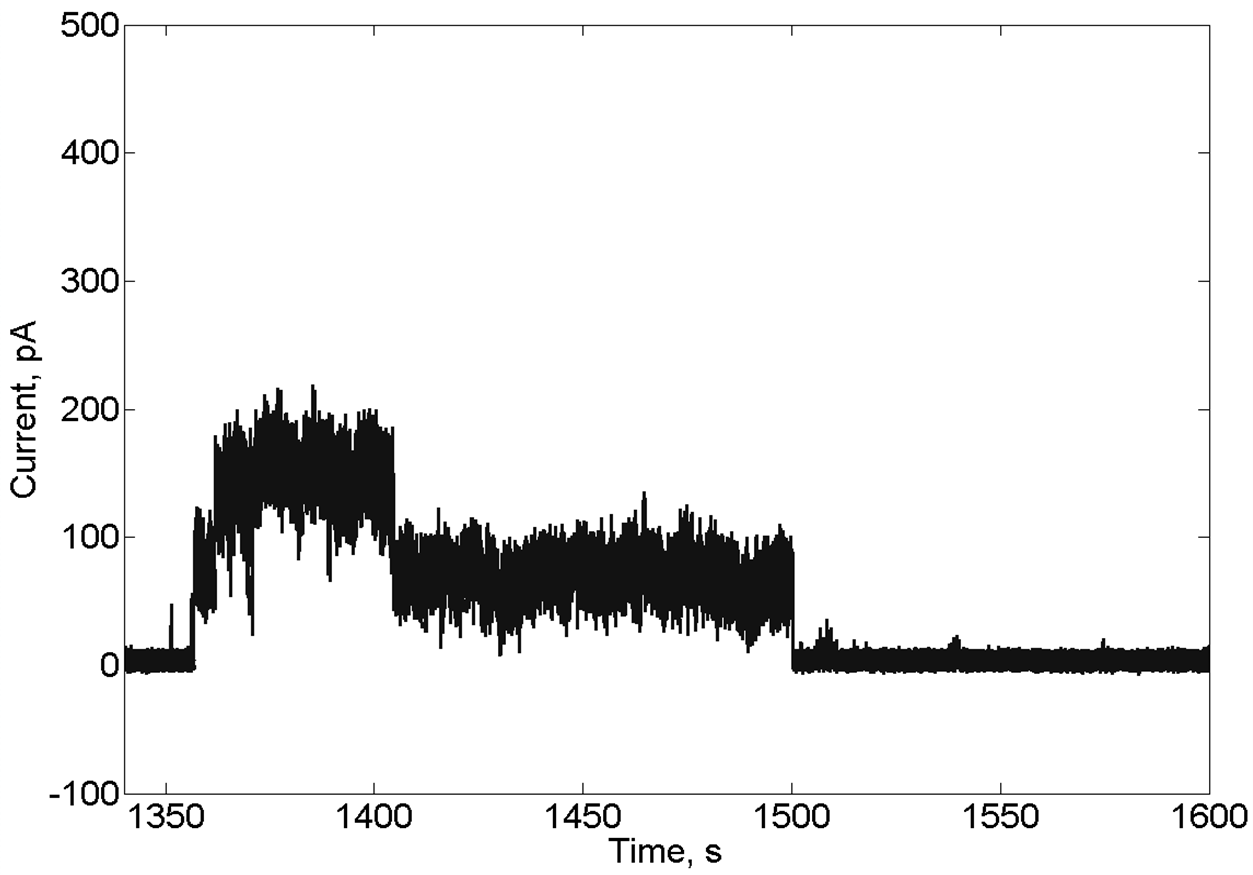


**Figure S1** Additional membrane current response at 84.7 nM cyt *c* demonstrates the transient pore formation in CL-containing lipid bilayer at the non-lytic porous state. Each droplet contains 50 mM KCl, 10 mM MOPS, pH 7.4 buffer; membrane composition is DPhPC/chol/CL = 55%/25%/20%; and voltage clamp: +50 mV.

**Figure S2** Model for the relationship between the free energy (E) of a pore and its radius (r) in a typical lipid bilayer. The free energy is evaluated in terms of kT; k is Boltzmann’s constant, T is the temperature. Evaluated for a lipid bilayer with λ=1×10^-11^ N and σ=2×10^-3^ N·m^-1^ at 300K.


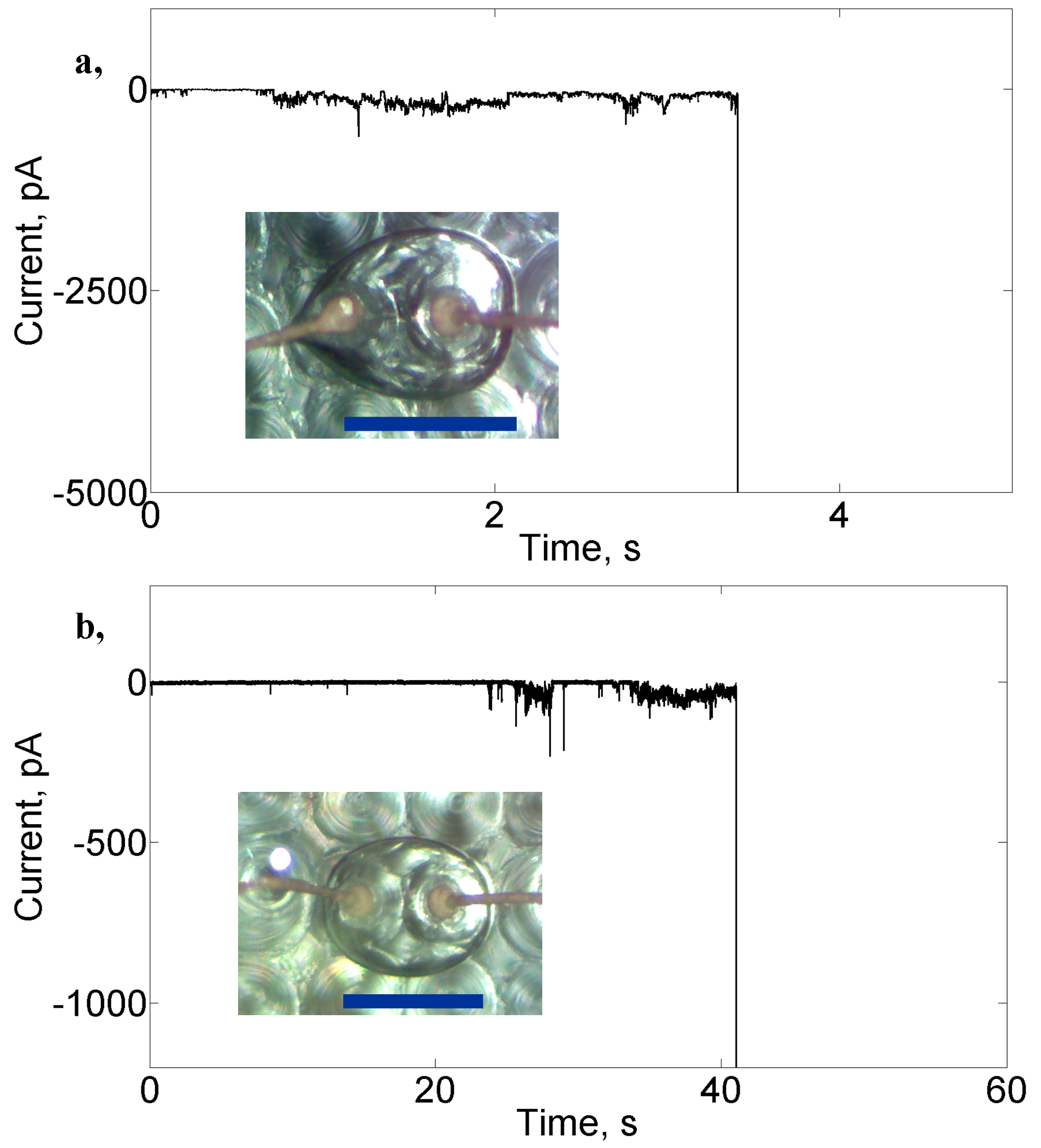


**Figure S3** Pore formation by the interaction between CL and PAMAM dendrimers. PAMAM dendrimers (G3) at concentrations of (a) 250nM and (b) 25μM can only induce the lytic porous state of permeabilization, while cyt *c* at these two concentrations induces the lytic porous and non-lytic highly permeable states respectively (Figure 2 and 4, main text). Buffer: 200 mM KCl, 10 mM MOPS, pH 7.4; membrane composition: DPhPC/chol/CL = 55%/25%/20%; voltage clamp: -50 mV.

## References

1. Zamzami N, Kroemer G (2001) The mitochondrion in apoptosis: how Pandora's box opens. Nature Reviews Molecular Cell Biology 2: 67-71.

2. Kagan VE, Bayir HA, Belikova NA, Kapralov O, Tyurina YY, et al. (2009) Cytochrome c/cardiolipin relations in mitochondria: a kiss of death. Free Radical Biology and Medicine 46: 1439-1453.

3. Belikova NA, Vladimirov YA, Osipov AN, Kapralov AA, Tyurin VA, et al. (2006) Peroxidase activity and structural transitions of cytochrome c bound to cardiolipin-containing membranes. Biochemistry 45: 4998-5009.

4. Northrup SH, Boles JO, Reynolds JC (1988) Brownian dynamics of cytochrome c and cytochrome c peroxidase association. Science 241: 67.

5. Zhu L, Wang K, Lu T, Xing W, Li J, et al. (2008) The direct electrochemistry behavior of Cyt c on the modified glassy carbon electrode by SBA-15 with a high-redox potential. Journal of Molecular Catalysis B: Enzymatic 55: 93-98.

6. Marini MA, Martin CJ, Berger RL, Forlani L (1974) A proposed solution for the determination of the ionization constants of sets of ionizing groups in proteins. Biopolymers 13: 891-902.

7. Hristova S, Zhivkov A, Atanasov B (2009) Electrostatics of horse heart cytochrome c and montmorillonite monolamellar plate. Biotechnology & Biotechnological Equipment 23: 568-571.

8. Korsmeyer SJ, Wei MC, Saito M, Weiler S, Oh KJ, et al. (2000) Pro-apoptotic cascade activates BID, which oligomerizes BAK or BAX into pores that result in the release of cytochrome c. Cell Death and Differentiation 7: 1166-1173.

9. Saito M, Korsmeyer SJ, Schlesinger PH (2000) BAX-dependent transport of cytochrome c reconstituted in pure liposomes. Nature Cell Biology 2: 553-555.

10. Baglioni P, Fratini E, Lonetti B, Chen SH (2004) Gelation in cytochrome C concentrated solutions near the isoelectric point: the anion role. Current opinion in colloid & interface science 9: 38-42.

11. Guo L, Pietkiewicz D, Pavlov EV, Grigoriev SM, Kasianowicz JJ, et al. (2004) Effects of cytochrome c on the mitochondrial apoptosis-induced channel MAC. American Journal of Physiology-Cell Physiology 286: C1109.

12. Tafani M, Karpinich NO, Hurster KA, Pastorino JG, Schneider T, et al. (2002) Cytochrome c release upon Fas receptor activation depends on translocation of full-length bid and the induction of the mitochondrial permeability transition. Journal of Biological Chemistry 277: 10073.

13. Bartoli CG, Pastori GM, Foyer CH (2000) Ascorbate biosynthesis in mitochondria is linked to the electron transport chain between complexes III and IV. Plant Physiology 123: 335.

14. Shimizu S, Narita M, Tsujimoto Y (1999) Bcl-2 family proteins regulate the release of apoptogenic cytochrome c by the mitochondrial channel VDAC. Nature 399: 483-487.

15. Pavlov EV, Priault M, Pietkiewicz D, Cheng EHY, Antonsson B, et al. (2001) A novel, high conductance channel of mitochondria linked to apoptosis in mammalian cells and Bax expression in yeast. The Journal of Cell Biology 155: 725.

16. Ow YLP, Green DR, Hao Z, Mak TW (2008) Cytochrome c: functions beyond respiration. Nature Reviews Molecular Cell Biology 9: 532-542.

17. Liu X, Kim CN, Yang J, Jemmerson R, Wang X (1996) Induction of apoptotic program in cell-free extracts: requirement for dATP and cytochrome c. Cell 86: 147-157.

18. Skulachev VP (1998) Cytochrome c in the apoptotic and antioxidant cascades. FEBS Letters 423: 275-280.

19. Munoz-Pinedo C, Guio-Carrion A, Goldstein JC, Fitzgerald P, Newmeyer DD, et al. (2006) Different mitochondrial intermembrane space proteins are released during apoptosis in a manner that is coordinately initiated but can vary in duration. Proceedings of the National Academy of Sciences 103: 11573.

20. Scorrano L, Ashiya M, Buttle K, Weiler S, Oakes SA, et al. (2002) A distinct pathway remodels mitochondrial cristae and mobilizes cytochrome c during apoptosis. Developmental Cell 2: 55-67.

21. Youle RJ, Strasser A (2008) The BCL-2 protein family: opposing activities that mediate cell death. Nature Reviews Molecular Cell Biology 9: 47-59.

22. Shimizu S, Ide T, Yanagida T, Tsujimoto Y (2000) Electrophysiological study of a novel large pore formed by Bax and the voltage-dependent anion channel that is permeable to cytochrome c. Journal of Biological Chemistry 275: 12321.

23. Schlame M, Brody S, Hostetler KY (1993) Mitochondrial cardiolipin in diverse eukaryotes. European Journal of Biochemistry 212: 727-733.

24. Pangborn MC (1942) Isolation and purification of a serologically active phospholipid from beef heart. Journal of Biological Chemistry 143: 247.

25. Ardail D, Privat JP, Egret-Charlier M, Levrat C, Lerme F, et al. (1990) Mitochondrial contact sites. Lipid composition and dynamics. Journal of Biological Chemistry 265: 18797.

26. Van Meer G, Voelker DR, Feigenson GW (2008) Membrane lipids: where they are and how they behave. Nature Reviews Molecular Cell Biology 9: 112-124.

27. Brdiczka D, Beutner G, Ruck A, Dolder M, Wallimann T (1998) The molecular structure of mitochondrial contact sites. Their role in regulation of energy metabolism and permeability transition. Biofactors 8: 235-242.

28. Kagan VE, Tyurin VA, Jiang J, Tyurina YY, Ritov VB, et al. (2005) Cytochrome c acts as a cardiolipin oxygenase required for release of proapoptotic factors. Nature Chemical Biology 1: 223-232.

29. Garcia Fernandez M, Troiano L, Moretti L, Nasi M, Pinti M, et al. (2002) Early changes in intramitochondrial cardiolipin distribution during apoptosis. Cell Growth and Differentiation 13: 449-455.

30. Haines TH, Dencher NA (2002) Cardiolipin: a proton trap for oxidative phosphorylation. FEBS Letters 528: 35-39.

31. Kates M, Syz JY, Gosser D, Haines TH (1993) pH-dissociation characteristics of cardiolipin and its 2 -deoxy analogue. Lipids 28: 877-882.

32. Heimburg T, Marsh D (1995) Protein surface-distribution and protein-protein interactions in the binding of peripheral proteins to charged lipid membranes. Biophysical Journal 68: 536-546.

33. Gorbenko GP, Molotkovsky JG, Kinnunen PKJ (2006) Cytochrome c interaction with cardiolipin/phosphatidylcholine model membranes: effect of cardiolipin protonation. Biophysical Journal 90: 4093-4103.

34. Brown LR, Wuthrich K (1977) NMR and ESR studies of the interactions of cytochrome c with mixed cardiolipin-phosphatidylcholine vesicles. Biochimica et Biophysica Acta (BBA)-Biomembranes 468: 389-410.

35. Birrell GB, Griffith OH (1976) Cytochrome c induced lateral phase separation in a diphosphatidylglycerol-steroid spin-label model membrane. Biochemistry 15: 2925-2929.

36. De Kruijff B, Cullis PR (1980) Cytochrome c specifically induces non-bilayer structures in cardiolipin-containing model membranes. Biochimica et Biophysica Acta (BBA)-Biomembranes 602: 477-490.

37. Seddon JM (1990) Structure of the inverted hexagonal (HII) phase, and non-lamellar phase transitions of lipids. Biochimica et Biophysica Acta 1031: 1.

38. Powell GL, Knowles PF, Marsh D (1990) Incorporation of cytochrome oxidase into cardiolipin bilayers and induction of nonlamellar phases. Biochemistry 29: 5127-5132.

39. Bayley H, Cronin B, Heron A, Holden MA, Hwang WL, et al. (2008) Droplet interface bilayers. Molecular Biosystems 4: 1191-1208.

40. Renner S, Geltinger S, Simmel FC Nanopore Translocation and Force Spectroscopy Experiments in Microemulsion Droplets. Small 6: 190-194.

41. Sarles SA, Leo DJ (2010) Regulated Attachment Method for Reconstituting Lipid Bilayers of Prescribed Size within Flexible Substrates. Analytical Chemistry 82: 959-966.

42. Sarles SA, Leo DJ (2010) Physical encapsulation of droplet interface bilayers for durable, portable biomolecular networks. Lab on a Chip 10: 710-717.

43. Mueller P, Rudin DO, Tien HT, Wescott WC (1963) Methods for the formation of single bimolecular lipid membranes in aqueous solution. Journal of Physical Chemistry 67: 534-535.

44. Mueller P, Rudin DO, Tien HT, Wescott WC (1962) Reconstitution of cell membrane structure in vitro and its transformation into an excitable system. Nature 194: 979-980.

45. Montal M, Mueller P (1972) Formation of bimolecular membranes from lipid monolayers and a study of their electrical properties. Proceedings of the National Academy of Sciences of the United States of America 69: 3561-3566.

46. Niles WD, Levis RA, Cohen FS (1988) Planar bilayer membranes made from phospholipid monolayers form by a thinning process. Biophysical Journal 53: 327-335.

47. Hanke W, Schlue W (1993) Planar lipid bilayers: methods and applications. San Diego, CA: Academic Press Inc.

48. Tien HT, Ottova-Leitmannova A (2003) Planar lipid bilayers (BLMs) and their applications: Elsevier Science.

49. Mountz JD, Ti Tien H (1978) Bilayer lipid membranes (BLM): study of antigen-antibody interactions. Journal of Bioenergetics and Biomembranes 10: 139-151.

50. Gray JR (2005) Conductivity Analyzers and Their Application. In: Down RD, Lehr JH, editors. Environmental Instrumentation and Analysis Handbook. Hoboken, NJ, USA: John Wiley & Sons, Inc. pp. 491-510.

51. Bockris JOM, Reddy AKN (2000) Modern electrochemistry: Springer Us.

52. Gallez D, Costa Pinto NM, Bisch PM (1993) Nonlinear dynamics and rupture of lipid bilayers. Journal of Colloid and Interface Science 160: 141-148.

53. Winterhalter M, Helfrich W (1987) Effect of voltage on pores in membranes. Physical Review A 36: 5874-5876.

54. Sung W, Park PJ (1997) Dynamics of pore growth in membranes and membrane stability. Biophysical Journal 73: 1797-1804.

55. Winterhalter M (2000) Black lipid membranes. Current Opinion in Colloid and Interface Science 5: 250-255.

56. Diederich A, Bahr G, Winterhalter M (1998) Influence of surface charges on the rupture of black lipid membranes. Physical Review E 58: 4883.
